# Depolarization imaging for fast and non-invasive monitoring of cervical microstructure remodeling in vivo during pregnancy

**DOI:** 10.1038/s41598-022-15852-w

**Published:** 2022-07-19

**Authors:** Jean Rehbinder, Jérémy Vizet, Junha Park, Razvigor Ossikovski, Jean-Charles Vanel, André Nazac, Angelo Pierangelo

**Affiliations:** 1 LPICM, CNRS, Ecole polytechnique, IP Paris, 91128 Palaiseau, France; 2 ICube, CNRS, Université de Strasbourg, 67412 Illkirch Cedex, France; 3 Department of Gynaecology, Iris Sud Ixelles Hospital, 1050 Ixelles, Belgium

**Keywords:** Biomedical engineering, Medical imaging

## Abstract

The cervix plays a crucial role in conception, maintenance of pregnancy, and childbirth. The mechanical properties of a pregnant woman's cervix change dramatically during gestation due to a remodeling of its microstructure, necessary for delivery. However, external factors can accelerate this process and lead to prematurity, the primary cause of perinatal mortality worldwide, due to the inefficiency of existing diagnostic methods. This study shows that polarized light is a powerful tool to probe the cervical microstructure during pregnancy. A wide-field multispectral polarimetric imaging system was fabricated to explore in vivo the cervix of full-term pregnant women. The polarimetric properties of the cervix change significantly with pregnancy progression. In particular, a set of several depolarization parameters (intrinsic and extrinsic) showed a strong linear correlation with gestational age in the red part of the visible spectral range. This trend can be attributed, among other things, to a decrease in collagen density and an increase in hydration of cervical connective tissue. Wide field depolarization imaging is a very promising tool for rapid and non-invasive analysis of cervical tissue in vivo to monitor the steady progression of pregnancy, providing the practitioner with useful information to improve the detection of preterm birth.

## Introduction

Prematurity is defined as birth at less than 37 weeks of amenorrhea and is the leading cause of perinatal mortality worldwide. Its rate is estimated at 6% in France and Europe and twice as high in the United States^1^. The threat of preterm birth (TPB) is the most important complication during pregnancy. It occurs between 23 and 37 weeks of amenorrhea and is characterized by uterine contractions associated with changes in the cervix's shape and sometimes bleeding or early rupture of the membranes. Women diagnosed with TPB are hospitalized and given tocolytic treatments to reduce contractions and suppress premature labor^2^. However, assessing TPB severity to predict preterm birth remains a difficult task in current medical practice^3^. The recommended technique is the transvaginal ultrasound for cervical length measurement, sometimes supplemented by biomarker detection^4,5^. Pregnant women with a cervical length of fewer than 20 mm at less than 37 weeks of amenorrhea are likely to deliver prematurely. However, because of the inability to correlate measured length with time to delivery, there is no clear recommendation for a threshold of the cervical length that would reliably detect prematurity^6^. Therefore, preterm delivery only occurs in less than 50% of women diagnosed with TPB, depending on the gestational age at the first admission^7^. Thus, a large percentage of women are unnecessarily hospitalized, which is costly^8^ and can lead to complications due to the side effects of tocolytic treatments^9^ and thromboembolic risks associated with bed rest^10^. A new method to improve prematurity diagnosis is needed to reduce healthcare costs, avoid adverse events related to unnecessary hospitalizations, and pave the way for new treatment strategies to decrease the preterm birth rate.

The cervix plays a crucial role in maintenance of pregnancy and childbirth^11^. The mechanical properties of the cervix dramatically change as gestation progresses. This process occurs slowly up to about 37 weeks of amenorrhea in a full-term pregnancy, resulting in a gradual softening of the cervix without any visible change in its anatomy (cervical softening), thus keeping the growing fetus in the uterus. After 37 weeks of amenorrhea, the cervix begins to shorten and dilate in preparation for labor and delivery (cervical ripening) which occurs at approximately 41 weeks of amenorrhea^12^. The change in mechanical properties of the cervix during pregnancy is due to the remodeling of its microstructure. This process mainly involves the connective tissue or stroma, accounting for more than 95% of the cervix's volume. It comprises 80% collagen (70% type I and 30% type II) with associated ground substance, 15% smooth muscle tissue, and 0.9% to 1.6% elastin^13^. Many studies have shown that connective tissue remodeling during pregnancy is a complex process involving an increase in collagen extractability and solubility, as well as a reduction in its concentration and organization^14–18^. In addition, an increase in collagenolytic activity and hydration of cervical tissue is also observed^17,18^. Thus, characterizing cervical microstructure remodeling during full-term gestation is crucial for understanding how this process unfolds for a high-risk pregnancy.

Different imaging techniques, such as X-ray diffraction^19^, optical coherence tomography (OCT)^20^, magnetic resonance imaging^21^, and second harmonic generation (SHG) microscopy^22^, have been used to study this remodeling process. However, the clinical application of these techniques during pregnancy remains limited. Indeed, X-rays can be potentially dangerous for the mother and the child. Furthermore, the side effects on the child of high magnetic fields are unknown^23^. Finally, OCT and SHG microscopy can probe the cervical tissue with micrometer resolution but to a maximum depth of less than one millimeter. In addition, they require a scanning system to image large areas, which makes these techniques slow and unsuitable for use in vivo.

Therefore, an innovative and safe tool is needed to quickly image a pregnant woman's cervix in vivo with a macroscopic field of view while providing information on its microstructure.

Polarimetric imaging has proven to be relevant for the characterization of highly scattering^24–26^ or anisotropic^27–29^ media. In recent years, this technique has shown great promise for various types of applications, including biomedical diagnosis^30–32^. Indeed, it has been widely used for the exploration of biological tissues, optically very complex systems where scattering and anisotropy effects are present at the same time. Polarimetric imaging is a technique using the polarization of light, which is highly sensitive to microstructural changes in biological tissues generated by pathological conditions. Indeed, it has shown great promise in detecting alterations in cell structure and density, as well as in the organization and size of fibers composing the extracellular matrix^33–36^. In particular, this technique is well suited to explore the structure of collagen, whose anisotropic and scattering properties can produce specific polarimetric signatures^36–38^. For this reason, it has been widely used to explore the microstructure of the cervix, which is rich in collagen^36,38–41^. Furthermore, it is a non-contact technique that can be implemented with a macroscopic field of view (several tens of cm^2^), while providing polarization contrasts sensitive to the sample microstructure at a scale much smaller than the actual image spatial resolution. In addition, it makes use of conventional light sources (LEDs, halogen lamps and others) and inexpensive optics. Continuous improvement in the design and construction of polarimeters has recently led to fast and stable systems well adapted for clinical applications^42–44^.

This article describes the results of a feasibility study whose main objective is to determine whether polarimetric imaging is an appropriate technique to characterize microstructural changes in the cervix during pregnancy. The study was conducted on a group of full-term pregnant women with different gestational ages, which allowed the identification of the potentially most relevant polarimetric parameters for monitoring the steady progression of a pregnancy.

## Results

Mueller polarimetric imaging was used in this study to obtain the comprehensive polarimetric characterization of the cervical tissue during pregnancy. This approach requires the measurement of the cervix's Mueller matrix ${\mathbf{M}}$, a real 4 × 4 matrix containing all its main polarimetric properties^45^. Different wavelengths in the visible spectral range were used to explore the cervical tissue at different depths, as explained in more detail below. For this purpose, we fabricated a multispectral Mueller Polarimetric Colposcope (MPC), enabling the fast and non-invasive examination of cervical tissue in vivo. It is an improved version of the first MPC prototype described in our previous publication^42^. The main novelty of this improved system lies in its ability to perform Mueller polarimetric imaging simultaneously at 550 nm and 650 nm.

The MPC was used to analyze the cervix of pregnant women of different gestational ages in hospital settings. This study was proposed to pregnant women over 14 weeks of amenorrhea without any exclusion criteria (age, medical history, ethnicity, and others) other than gestational age, resulting in a very inhomogeneous population.

The main goal of this study was to determine the most relevant polarimetric parameters to monitor the steady remodeling of the cervical microstructure during pregnancy. For this reason, only women who delivered at term were considered for the final analysis.

After polarimetric imaging, cervical length was also measured by transvaginal ultrasound (conventional procedure) for comparison. The "Methods and materials" section details the experimental setup for data acquisition in vivo, the clinical protocol, and data analysis.

### Comprehensive polarimetric characterization of cervical tissue for pregnant women

The cervix is the uterus's lower part protruding into the vagina. It has a cylindrical shape and is crossed for its entire length by the endocervical canal connecting the vaginal lumen to the uterine cavity. The entrance to the endocervical canal on the vaginal side is called the "external os". The other end of the endocervical canal on the side of the uterine cavity is called the "internal os".

The practitioner used the MPC like a conventional colposcope to characterize the polarimetric response of the ectocervix, the part of the cervix located at the interface with the vaginal cavity. As a first step, the Mueller matrix ${\mathbf{M}}$ obtained at a specific wavelength for each patient's cervix was processed using the symmetric decomposition, a nonlinear algebraic compression algorithm described in detail by Eq. (3) in the "Methods and materials" section, to determine the main polarimetric properties of cervical tissue, namely the depolarization and the linear birefringence. Other polarimetric properties such as circular birefringence and diattenuation were found to be negligible. Then, the total depolarization ∆ at 550 nm and 650 nm was calculated for each pixel using Eq. (4). This parameter quantifies the depolarizing power of the cervical tissue for linearly and circularly polarized light. The linear birefringence is characterized by the linear phase retardance ${\text{R}}$ and the azimuth ${\upalpha }$, calculated for each pixel using Eqs. (5) and (6), respectively. The parameter ${\text{R}}$ quantifies the difference in optical phase shifts between two polarization eigenstates, called the fast and slow axis. The parameter ${\upalpha }$ gives the direction of the slow axis projected onto the image plane.

**Figure 1:**
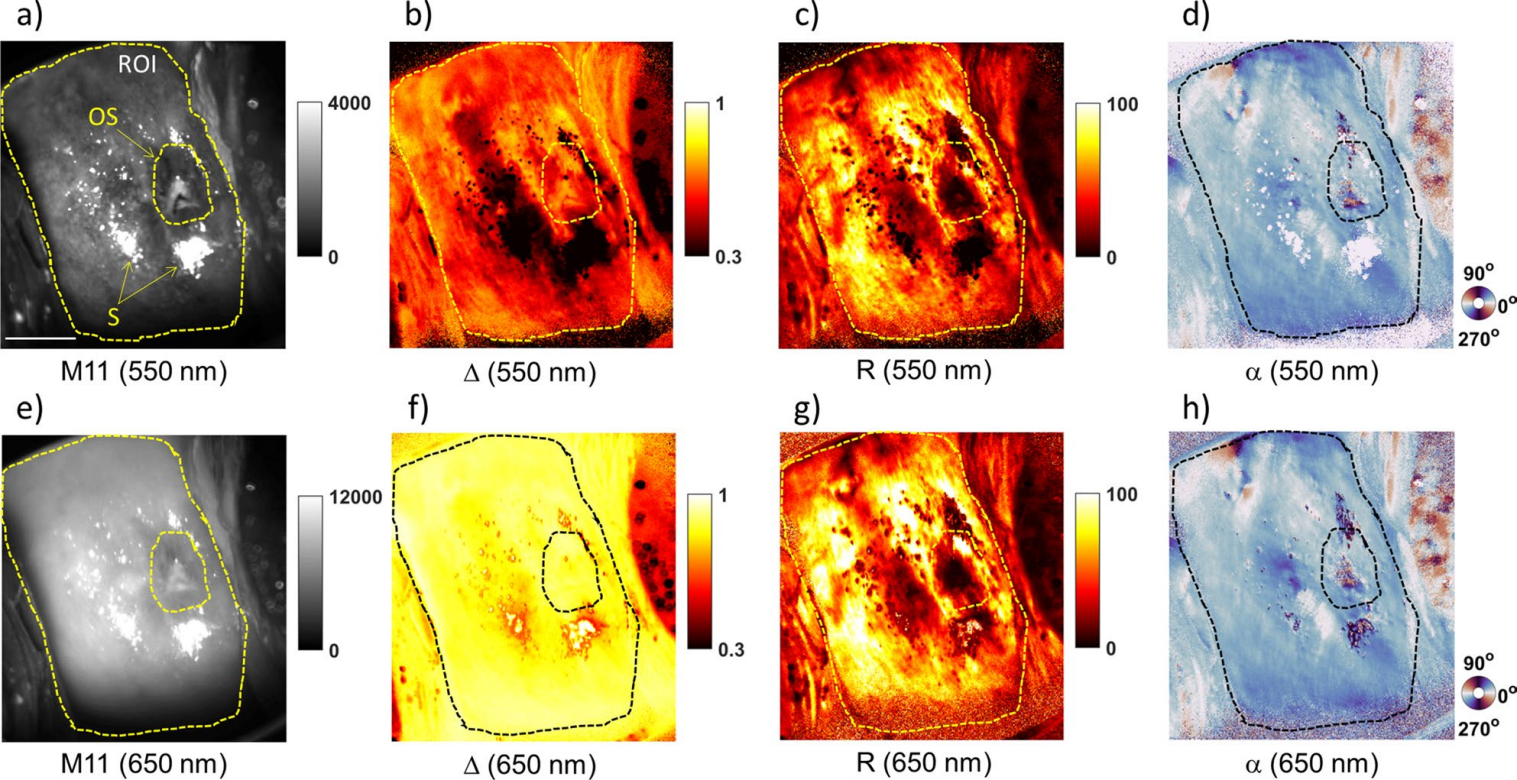
**(a**, **e**), Monochromatic unpolarized intensity image of patient P1's ectocervix corresponding to the unnormalized coefficient ${\text{M}}_{11}$ of the measured Mueller matrix at 550 and 650 nm, respectively. (**b**, **f**) Total depolarization image $\Delta$ at 550 and 650 nm, respectively. (**c**, **g**) Linear phase retardance image ${\text{R}}$ at 550 and 650 nm, respectively. (**d**, **h**) Azimuth image ${\upalpha }$ at 550 and 650 nm, respectively. In (**a**), the white bar corresponds to 1 cm, OS indicates the pixels in the region of the external os, and S indicates the pixels saturated by specular reflections.

Figure 1 shows, as an example, the images obtained for a pregnant woman's cervix at 29 weeks of amenorrhea and 3 days, referred to as P1 in the text below. The image of the non-normalized backscattered light intensity (${\text{M}}_{11}$ coefficient of the measured Mueller matrix) is presented in Fig. 1a for 550 nm and in Fig. 1e for 650 nm. Figure 1b–d show the $\Delta$, ${\text{R}}$ and ${\upalpha }$ images, respectively, at 550 nm. Figure 1f–h show the same parameters at 650 nm. The absorption of the cervical tissue is overall different for the two wavelengths considered, the ${\text{M}}_{11}$ image being spatially more homogeneous at 650 nm than at 550 nm, as shown in Fig. 1a,e. The $\Delta$ image is also characterized by a higher spatial homogeneity at 650 nm than at 550 nm, as shown in Fig. 1b,f. On the contrary, the spatial pattern of the ${\text{R}}$ image is very similar for the two wavelengths considered, as shown in Fig. 1c,g. The same applies to the ${\upalpha }$ image, as shown in Fig. 1d,h.

**Figure 2:**
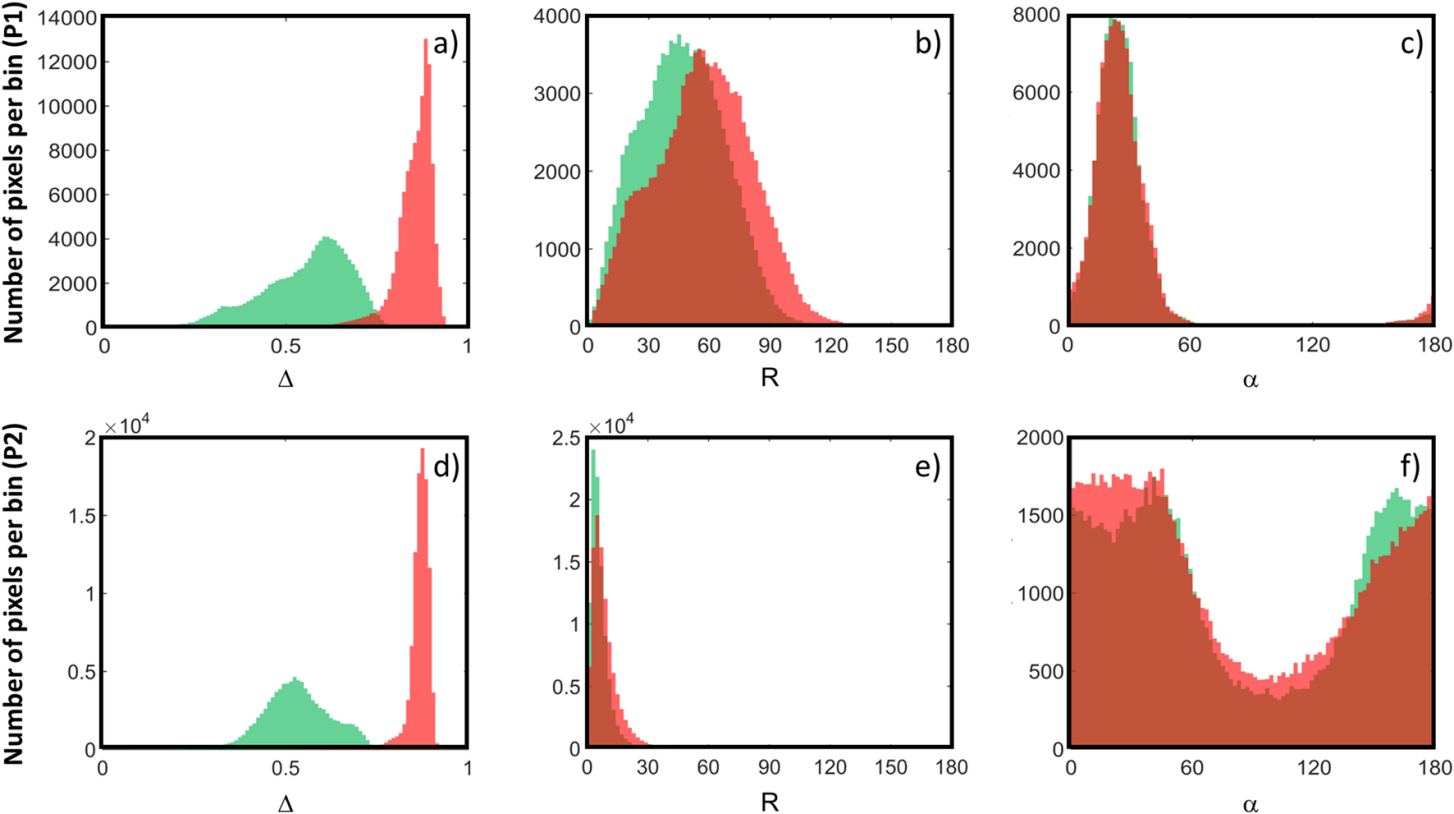
**(a**, **d**), The histograms of the ∆ images obtained for P1 and P2, respectively. (**b**, **e**) The histograms of the R images obtained for P1 and P2, respectively. (**c**, **f**) The histograms of the ${\upalpha }$ images obtained for P1 and P2, respectively. All the histograms are calculated for the pixels in the selected ROIs (the green and red color corresponding to 550 and 650 nm, respectively).

To quantitatively compare the $\Delta$, ${\text{R}}$ and ${\upalpha }$ images at 550 nm and 650 nm, a region of interest (ROI) outlining the ectocervix is selected for P1, as shown in Fig. 1. All pixels saturated by specular reflections (indicated by S in Fig. 1a) are excluded from the ROI. The same applies to the pixels in the region of the external os (indicated by OS in Fig. 1a). The histograms of $\Delta$, ${\text{R}}$ and ${\upalpha }$ images, calculated for all pixels of the selected ROI, are shown in Fig. 2a–c, respectively. The results described below have been obtained when comparing $\Delta$, ${\text{R}}$ and ${\upalpha }$ images of P1 at 550 nm and 650 nm.

- The overlap between the histograms of $\Delta$ at 550 nm and 650 nm is negligible. In particular, the value of $\Delta$ is significantly higher overall at 650 nm than at 550 nm. In addition, the histogram of $\Delta$ is more spread out at 550 nm than at 650 nm.
- The histograms of ${\text{R}}$ at 550 nm and 650 nm overlap strongly. Specifically, the value of ${\text{R}}$ for each pixel is slightly higher at 650 nm than at 550 nm. In contrast, the histograms of ${\upalpha }$ for the two wavelengths are almost totally overlapping.

Similar results were obtained by comparing polarimetric images at 550 nm and 650 nm for the cervix of other patients, regardless of their gestational age.

In addition, the polarimetric images of P1 were compared with those obtained for a second pregnant woman of approximately the same gestational age, i.e., at 28 weeks of amenorrhea and 5 days, to assess the inter-patient variability. For completeness, the images obtained for this patient, referred to as P2 in the text below, are shown in Fig. S1 (Appendix). A ROI is also selected for P2, as shown in Fig. S1. For a quantitative comparison between the two patients, the histograms of $\Delta$, ${\text{R}}$ and ${\upalpha }$ images of P2 at 550 nm and 650 nm are shown in Fig. 2d–f, respectively. Similar results to those previously described for P1 are also obtained for P2 when comparing the polarimetric properties of this patient's cervix at 550 nm and 650 nm. In addition, the results described below have been obtained when comparing $\Delta$, ${\text{R}}$ and ${\upalpha }$ images of P1 and P2 for both wavelengths.

- The histogram of $\Delta$ at 650 nm is quite narrow and centered around a peak value that is very similar for P1 and P2 (about 1% of difference). In contrast, the histogram of $\Delta$ at 550 nm is much more spread out than at 650 nm for P1 and P2. For each patient, the value of this parameter for most pixels varies around a peak value that is significantly different between P1 and P2 (more than 10% difference).
- The histograms of ${\text{R}}$ and ${\upalpha }$ are very different between P1 and P2 for both wavelengths. In particular, the histogram of ${\text{R}}$ is much more widespread for P1 than for P2. In addition, the peak value of ${\text{R}}$ is significantly higher for P1. The histogram of ${\upalpha }$ is narrow for P1 and shows a peak around about the same value at 550 nm and 650 nm. On the contrary, it is spread out between 0° and 180° for P2 with no specific peak value for both wavelengths.

The ranges of variability (in brackets) and the peak values (in bold) of the $\Delta$, ${\text{R}}$ and ${\upalpha }$ parameters for P1 and P2 at 550 nm and 650 nm are summarized in Table 1.

**Table 1:** Ranges of variability between the 5th and 95th percentile (in brackets) and the maximum values (in bold) of $\Delta$, ${\text{R}}$ and ${\upalpha }$ for P1 and P2 at 550 nm and 650 nm.

	Δ		R		$\upalpha$	
	P1	P2	P1	P2	P1	P2
550 nm	(0.31–0.72) **0.61**	(0.39–0.69) **0.53**	(13.6°–81.1°) **45.2**°	(1.2°–14.5°) **3.6**°	(8.9°–47.9°) **21.3**°	(0°–180°) **No peak**
650 nm	(0.75–0.91) **0.89**	(0.81–0.90) **0.88**	(16.7°–96.1°) **56.6°**	(1.7°–21.5°) **5.0°**	(8.3°–54.2°) **23.0°**	(0°–180°) **No peak**

Similar results to those obtained by comparing P1 and P2, which have been described in detail as illustrative examples, were also observed by comparing other groups of patients with approximately the same gestational age, regardless of the pregnancy stage. As a general trend, the ${\text{R}}$ and ${\upalpha }$ parameters are characterized by a high inter-patient variability for both wavelengths. This inter-patient variability is also high for $\Delta$ at 550 nm while it becomes significantly lower for $\Delta$ at 650 nm. In the following paragraph, the inter-patient variability will be discussed in a more quantitative way.

### Evolution of the main cervical polarimetric properties according to the gestational age

In this section, the evolution of the main cervical polarimetric properties according to the gestational age is investigated. For each patient, the mean values of $\Delta$ and ${\text{R}}$, denoted as $\mu \left( \Delta \right)$ and $\mu \left( {\text{R}} \right),$ respectively, are calculated at 550 nm and 650 nm for all pixels contained in a ROI selected with the procedure described previously. A different approach is used for ${\upalpha }$. Indeed, this parameter determines the spatial orientation of the ordered microscopic structures that generate ${\text{R}}$. The absolute value of ${\upalpha }$ for each pixel depends on the orientation of the cervix, which varies considerably from patient to patient. Thus, the Kurtosis $k$ of ${\upalpha }$ is calculated over a 3 × 3 pixel window around each pixel of the selected ROI to obtain a parameter independent of cervical orientation to determine the degree of the tissue's local microscopic organization. Then, the average value of $k$, denoted as $\mu \left( k \right)$, is calculated for all pixels of the selected ROI for both wavelengths. Finally, the evolution curves of $\mu \left( \Delta \right)$, $\mu \left( {\text{R}} \right)$, and $\mu \left( k \right)$ according to the gestational age are plotted.

**Figure 3:**
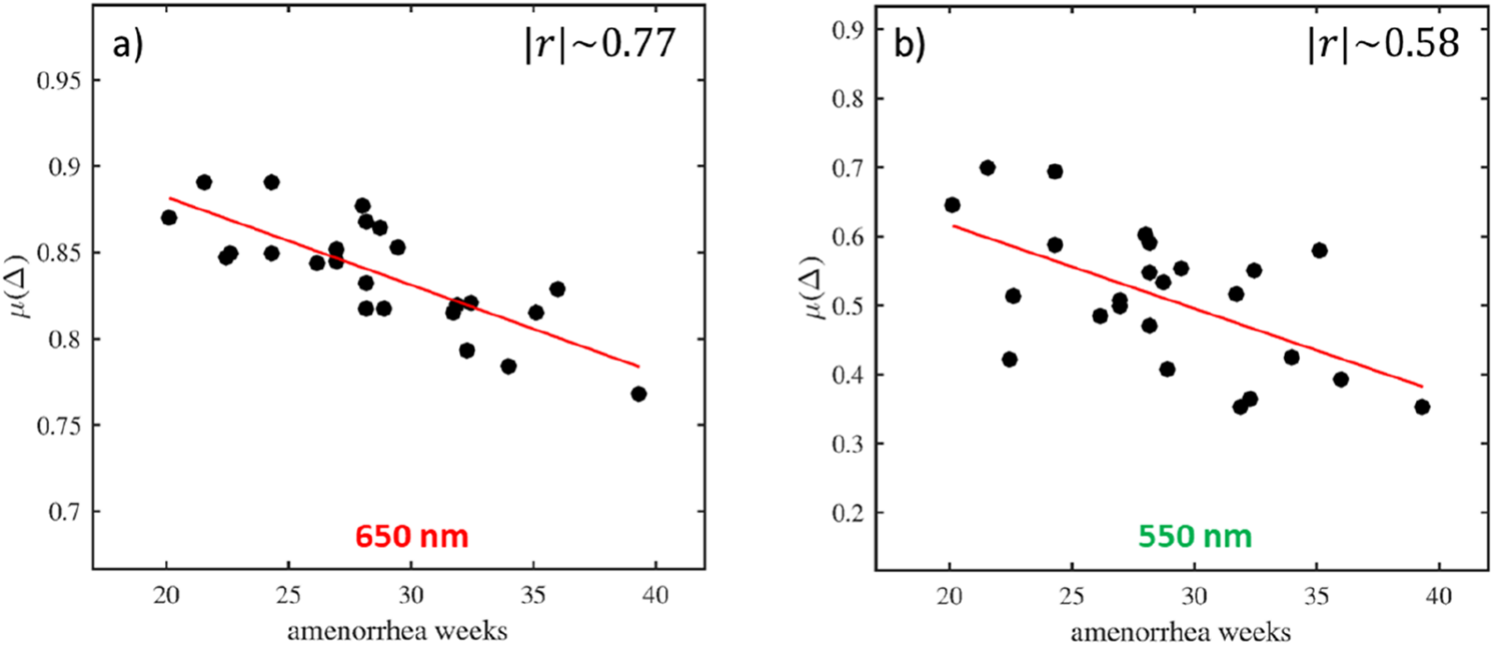
**(a**, **b**) Evolution curves describing the modification of ${\upmu }\left( \Delta \right)$ according to the gestational age at 650 nm and 550 nm, respectively. A negative linear correlation is observed between ${\upmu }\left( \Delta \right)$ and gestational age for both wavelengths. This correlation is high at 650 nm $\left( {\left| {\text{r}} \right|\sim 0.77} \right)$ and moderate at 550 nm $\left( {\left| {\text{r}} \right|\sim 0.58} \right)$.

A negative linear correlation is observed between $\mu \left( \Delta \right)$ and the gestational age for both wavelengths. This correlation is high at 650 nm, with an absolute value for the Pearson correlation coefficient given by $\left| r \right|\sim 0.77.$ Otherwise, it is moderate at 550 nm with $\left| r \right|\sim 0.58$. The evolution curves of $\mu \left( \Delta \right)$ at 650 nm and 550 nm are shown in Fig. 3a,b, respectively.

The parameters $\mu \left( {\text{R}} \right)$ and $\mu \left( k \right)$ are not correlated with the gestational age for both wavelengths. For completeness, the evolution curves of $\mu \left( {\text{R}} \right)$ and $\mu \left( k \right)$ are shown in Figs. S2 and S3 (Appendix), respectively.

**Figure 4:**
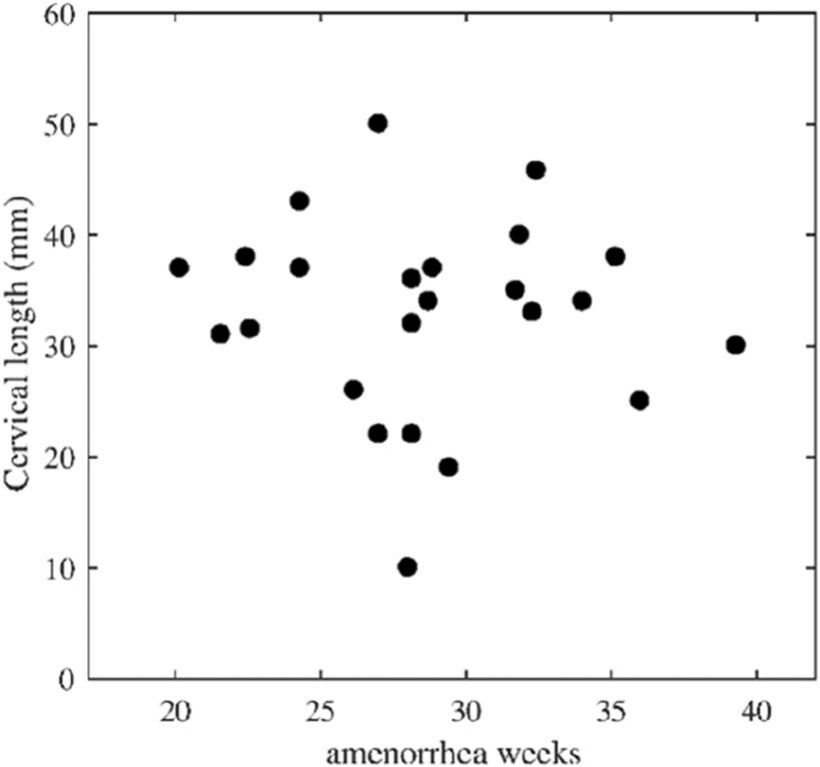
Evolution curve of the cervical length measured by transvaginal ultrasound (standard procedure) according to the gestational age. No correlation is observed between cervical length and gestational age.

Cervical length measured by transvaginal ultrasound is not correlated with gestational age. The evolution curve of the cervical length is shown in Fig. 4.

Overall, $\mu \left( \Delta \right)$ decreases linearly with gestational age for both wavelengths, which suggests that the total depolarization $\Delta$ may be a polarimetric parameter well suited to monitor the evolution of the cervical microstructure during pregnancy. However, the correlation of $\mu \left( \Delta \right)$ with the gestational age is high at 650 nm while it is moderate at 550 nm. This difference can be explained by the fact that the inter-patient variability for this parameter between two women with approximately the same gestational age remains quite low (up to a maximum of 5%) at 650 nm, while it can increase dramatically at 550 nm (up to a maximum of 30%), as can be seen in Fig. 3a,b.

On the contrary, $\mu \left( {\text{R}} \right)$ does not show any correlation with the gestational age and it is characterized by an inter-patient variability which can be very high, as shown in Fig. S2 (Appendix). The same applies to $\mu \left( k \right)$, as shown in Fig. S3 (Appendix). Consequently ${\text{R}}$ and ${\upalpha }$ parameters seem to be not suitable for monitoring the evolution of the cervical microstructure during pregnancy.

Finally the total depolarization $\Delta$, especially at 650 nm, appeared to be the most relevant and robust parameter that can potentially be used to monitor the steady pregnancy progression.

### Evaluation of complementary depolarization metrics

**Figure 5:**
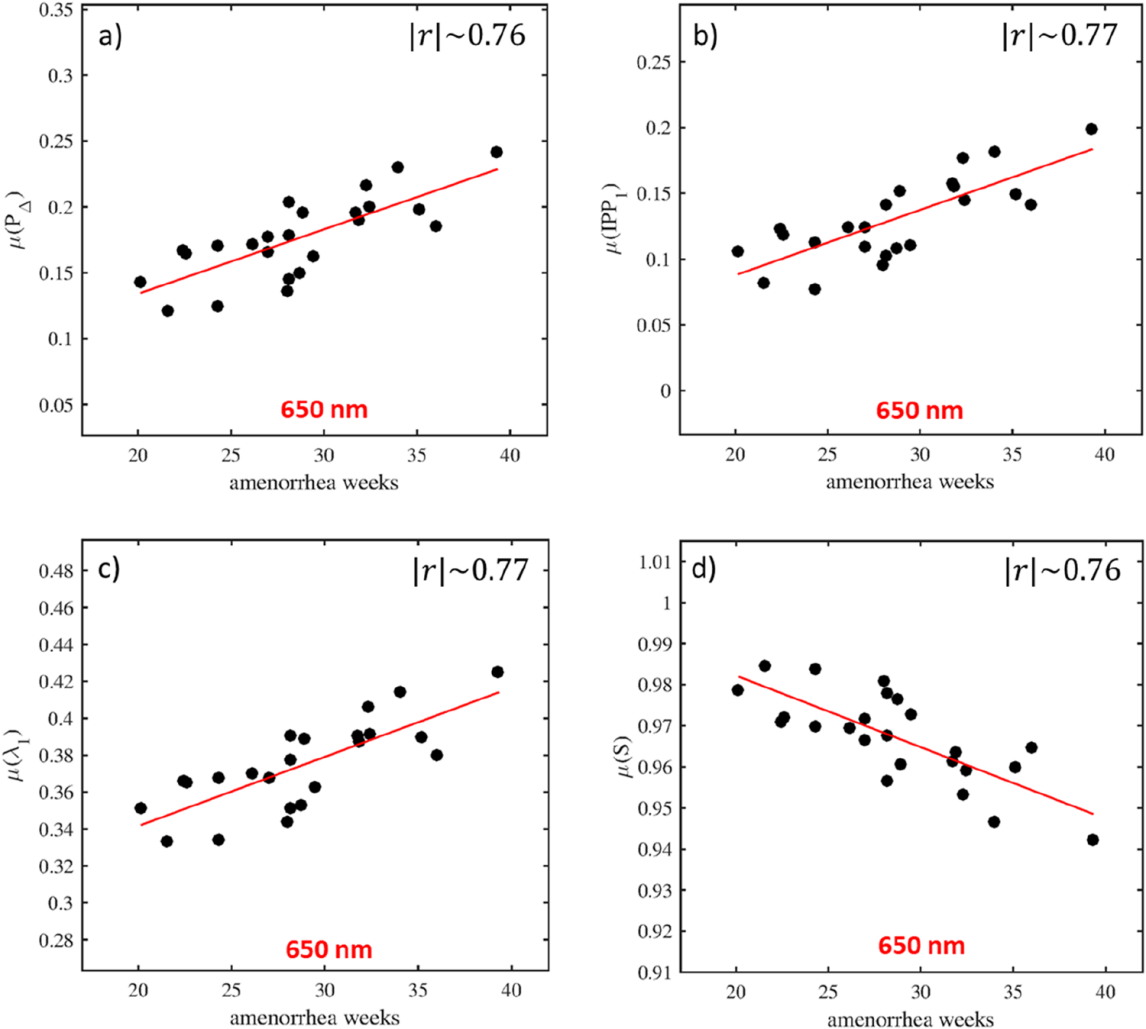
**(a**–**d**) Evolution curves describing the modification of ${\upmu }\left( {{\text{P}}_{\Delta } } \right)$, ${\upmu }\left( {{\text{IPP}}_{1} } \right)$, ${\upmu }\left( {{\uplambda }_{1} } \right)$, and ${\upmu }\left( {\text{S}} \right)$ at 650 nm according to the gestational age, respectively. These parameters have a high correlation with the gestational age, $\left| {\text{r}} \right|$ varying between $0.76$ and $0.77$.

Other depolarization metrics also exist that can be calculated from the measured Mueller matrices. In order to obtain a complete characterization of the depolarization properties of the cervix, these other depolarization metrics, described in detail in the Methods and Materials section, were also tested. These parameters are: the depolarization index ${\text{P}}_{\Delta }$ (Eq. 7), the eigenvalues ${\uplambda }_{1}$, ${\uplambda }_{2}$, ${\uplambda }_{3}$, and ${\uplambda }_{4}$ of the covariance matrix ${\text{H}}$ (Eq. 8), the polarimetric entropy ${\text{S}}$ (Eq. 9), the purity indices ${\text{IPP}}_{1}$, ${\text{IPP}}_{2}$, and ${\text{IPP}}_{3}$ (Eqs. 10–12), and the overall purity ${\text{PI}}$ (Eq. 13). In general, the curve describing the evolution of the mean value of each parameter as a function of the gestational age shows a linear trend, which can be explained by the negligible diattenuation of the cervical tissue. Indeed, the diattenuation makes the difference between this set of depolarization parameters directly calculated from the measured Mueller matrix ${\mathbf{M}}$ (called "extrinsic" parameters) and that obtained from the symmetric decomposition (called "intrinsic" parameters)^46^. In the absence of diattenuation, both sets of depolarization parameters coincide. However, only $\mu \left( {{\text{P}}_{\Delta } } \right)$, $\mu \left( {{\text{IPP}}_{1} } \right)$, $\mu \left( {{\uplambda }_{1} } \right)$, and $\mu \left( {\text{S}} \right)$ perform as well as $\mu \left( \Delta \right)$,as shown in Fig. 5 for 650 nm and in Fig. S4 (Appendix) for 550 nm. In particular, $\mu \left( {{\text{P}}_{\Delta } } \right)$, $\mu \left( {{\text{IPP}}_{1} } \right)$, $\mu \left( {{\uplambda }_{1} } \right)$ have a positive linear correlation with the gestational age for both wavelengths. On the contrary, this correlation is negative for $\mu \left( {\text{S}} \right)$. For these parameters, the correlation is high at 650 nm with $\left| r \right|$ varying between $0.76$ and $0.77$. The correlation is moderate at 550 nm with $\left| r \right|$ varying between $0.57$ and $0.6$.

Another interesting parameter is also $\mu \left( {{\text{PI}}} \right)$ at 650 nm, which performs slightly less well than $\mu \left( \Delta \right)$, but for which the correlation coefficient remains high ($\left| r \right|\sim 0.72$), as shown in Fig. S5a) (Appendix). The correlation is moderate at 550 nm for this parameter with $\left| r \right|\sim 0.56$, as shown in Fig. S5b) (Appendix).

For all the other parameters, i.e., $\mu \left( {{\text{IPP}}_{2} } \right)$, $\mu \left( {{\text{IPP}}_{3} } \right)$, $\mu \left( {{\uplambda }_{2} } \right)$, $\mu \left( {{\uplambda }_{3} } \right)$, and $\mu \left( {{\uplambda }_{4} } \right)$ the correlation is moderate for both wavelengths, with $\left| r \right|$ varying from a minimum of 0.49 to a maximum of 0.67, as shown in Figs. S6–S10 (Appendix).

## Discussion

The depolarization $\Delta$ allows the volume scattering properties of the cervix to be explored to a depth of a few hundred micrometers at 550 nm and of the order of a centimeter at 650 nm^47^. The difference in light penetration depth as a function of wavelength is mainly due to the hemoglobin absorption, which is much higher at 550 nm than at 650 nm. Thus, the light at 650 nm is more strongly scattered in the tissue due to its greater penetration depth, which explains the overall higher tissue depolarization observed at this wavelength. Furthermore, the hemoglobin concentration varies significantly from one area of the cervix to another, thus determining the strong spatial inhomogeneity of $\Delta$ at 550 nm, the most absorbing areas being also the least depolarizing. In contrast, the hemoglobin absorption is negligible at 650 nm, which explains the higher spatial homogeneity observed for $\Delta$ at this wavelength.

Surface scattering properties can also contribute to the spatial inhomogeneity observed for $\Delta$ at 550 nm due to the shallow depth of light penetration at this wavelength. Indeed, the surface morphology of the ectocervix can change significantly during pregnancy. In general, a stratified squamous epithelium (Malpighian epithelium), about 300 microns thick, lines the surface of the ectocervix. This type of epithelium helps protect the cervix from the high acidity of the vaginal environment. In contrast, a single layer of columnar mucous cells (glandular epithelium), 5–10 microns thick, lines the endocervical canal characterized by very low acidity. The connective tissue underlies these two epithelia. The boundary between the Malpighian epithelium and the glandular epithelium is the junction zone, usually placed around the external os. However, due to an increase in estrogen, the external os may open, making visible on the ectocervix the glandular epithelium (ectropion)^48^. Over time, due to the high acidity of the vagina, the glandular epithelium gradually changes into Malpighian epithelium better adapted to this type of environment (metaplasia)^48^. Thus, at the time of polarimetric image acquisition, areas of Malpighian epithelium, ectropion, and metaplasia, with very different scattering properties, are randomly distributed on the cervix surface depending on many factors such as parity, sexual activity, and others. For instance, for the two cases described before, patient P1's ectocervix was almost entirely covered by Malpighian epithelium, whereas patient P2's ectocervix consisted of randomly distributed areas of glandular epithelium and metaplasia.

The strong dependence of $\Delta$ at 550 nm on the scattering properties of the most superficial layers also contributes to the high inter-patient variability observed for this parameter. On the contrary, surface inhomogeneity has a much smaller impact on the measurement of $\Delta$ at 650 nm due to the longer light penetration depth at this wavelength. Therefore, this parameter provides information regarding the bulk scattering properties of the cervix. As $\Delta$ at 650 nm is spatially more homogeneous compared to 550 nm, this suggests that the bulk scattering of the cervix is much more homogeneous than that of the more superficial layers.

The hemoglobin concentration as well as the surface structure of the ectocervix changes during pregnancy and can differ significantly between two pregnant women of approximately the same gestational age, thus producing a high inter-patient variability for $\Delta$ at 550 nm but much lower for $\Delta$ at 650 nm.

The anisotropy-related ${\text{R}}$ image shows very similar spatial patterns when measured at 550 nm and 650 nm, even though the difference in light penetration depth is high between these two wavelengths. The same applies to ${\upalpha }$. This effect suggests that the ${\text{R}}$ and ${\upalpha }$ parameters are weakly affected by hemoglobin absorption. Moreover, they are mainly related to the anisotropy produced by the collagen fibers of the most superficial connective tissue layers, close to the epithelium^36,42^. In the case where the collagen fibers are well aligned and their orientation varies on a scale much larger than the image spatial resolution, they produce a high value of ${\text{R}}$ that increases with increasing collagen density. The ${\upalpha }$ parameter describes the orientation of the slow axis of the linear phase retardance and is parallel to the orientation of the collagen fibers projected onto the image plane^38^. The value of ${\text{R}}$ decreases significantly if the density of collagen fibers is low or if their orientation varies at a scale much smaller than the spatial resolution of the image. Depending on the type of epithelium covering the surface of the cervix, the degree of anisotropy of the underlying connective tissue can be significantly different^36^. Therefore, the morphology of the cervical surface has a strong impact on the spatial homogeneity of these parameters and is also at the origin of their high inter-patient variability observed between two pregnant women with approximately the same gestational age.

## Conclusion

This study shows that polarimetric imaging can be a very promising tool to probe the cervical microstructure during gestation.

A miniaturized Mueller polarimeter mounted on a conventional colposcope was used to examine the cervix of pregnant women of different gestational ages. The total depolarization ∆, obtained from the measured Mueller matrix using the symmetric decomposition, was found to be the most relevant parameter to monitor the cervical microstructure remodeling during pregnancy.

Indeed, the mean value μ(∆) of this parameter, calculated for all pixels contained in a ROI delineating the ectocervix, correlates linearly with gestational age at 550 nm and 650 nm. The physiological origin of the decrease in μ(∆) with the pregnancy progression for both wavelengths can be attributed to a decrease in the volume scattering power of the cervix mainly due to, among other things, a decrease in collagen density and an increase in connective tissue hydration at different depths^14–18^.

Among the two wavelengths tested, the one at 650 nm is the most suitable for exploring the cervix. Indeed, the ∆ parameter at 650 nm shows a smaller inter-patient variation than at 550 nm between two patients with approximately the same gestational age. For this reason, μ(∆) showed a high linear correlation with gestational age. Furthermore, despite the high heterogeneity of the population considered, this high correlation shows the robustness of this parameter and its strong potential to define a standardization curve.

Finally, the other depolarization parameters, in particular, ${\text{P}}_{\Delta }$, ${\text{IPP}}_{1}$, ${\uplambda }_{1}$, and ${\text{S}}$ at 650 nm showed a performance close to that observed for ∆ at the same wavelength. The great advantage of these parameters compared to ∆ is that they can be calculated directly from the measured Mueller matrix without applying nonlinear compression algorithms, such as the symmetric decomposition, which considerably reduce the propagation of numerical errors and computational time. Integrating the algorithms to calculate these parameters on the MPC can help develop a non-invasive and rapid diagnostic tool that can accurately assess the cervical microstructure in real-time during routine hospital use.

The results obtained in this study show that several depolarization parameters, particularly in the red/near-infrared portion of the visible spectrum, hold great promise for characterizing the microstructural changes of the cervix during pregnancy. This study has paved the way for a 36-month clinical study, currently underway, conducted on a massive number of patients that will be analyzed multiple times at different gestational ages. Our next goal will be to establish a standardization curve describing the steady progression of the pregnancy. Knowledge of the typical behavior of cervical depolarization properties during a full-term pregnancy could allow detection of abnormalities in their evolution and lead to a better diagnosis of prematurity.

## Methods and materials

### Experimental setup for data acquisition in vivo

**Figure 6:**
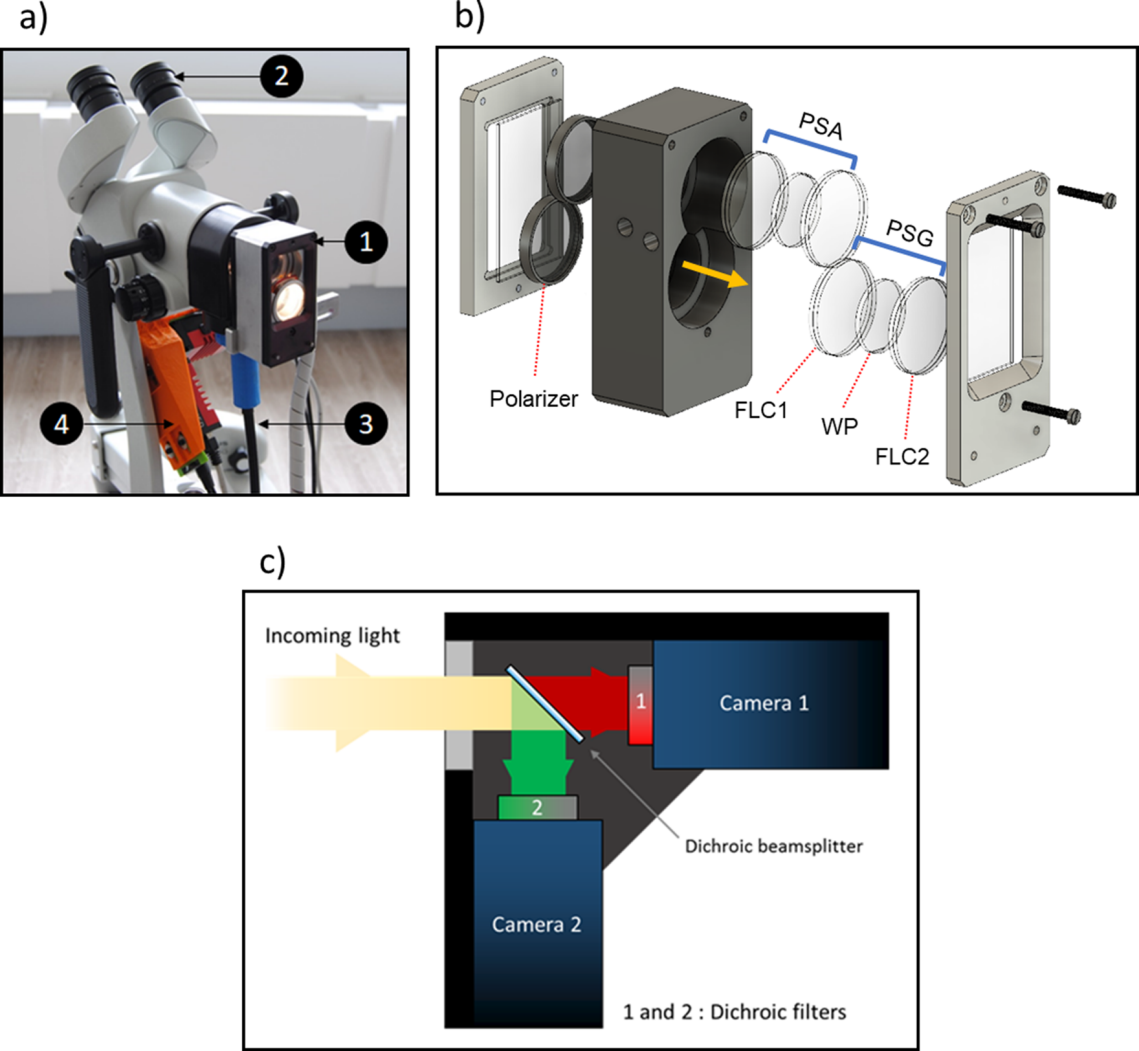
(**a**) Photo of the entire MPC (1: Mueller polarimeter, 2: binocular system for stereoscopic vision of the cervix, 3: liquid light guide, 4: dual-camera system). (**b**) Sketch of the polarimetric box and detailed description of PSG and PSA (image created with SOLIDWORKS Premium 2021 SP4.1, URL: http://www.3ds.com/terms/ost). The orange arrow indicates the direction of light propagation. (**c**) Incoming light from the optical output of the colposcope is guided to the dual-camera system, allowing the measurement of Mueller polarimetric images simultaneously at 650 nm (1) and 550 nm (2).

The MPC, shown in Fig. 6a, was obtained by modifying a commercial colposcope (Olympus OCS-500), routinely used for cervical analysis in established medical practice for cancer screening. The colposcope is a binocular stereoscopic microscope that allows direct illumination and observation of the cervix at low magnification (between 4 and 6X). Unpolarized light is supplied by a halogen source and transported to the colposcope head through a liquid light guide (Thorlabs LLG0528-6). A collimator placed at the exit tip of the liquid light guide enables to produce a beam with a spot size of 6.5 cm at a work distance of 30 cm from the colposcope head (corresponding approximately to the position of the cervix to analyze).

A home-made miniaturized Mueller polarimetric system, framed in a 30 × 50 × 100 mm^3^ sealed metal box, is placed in front of the colposcope on the patient side, as shown in Fig. 1a. The light exiting from the collimator passes through a Polarization State Generator (PSG) before illuminating the cervix. The light backscattered from the tissue is analyzed by a Polarization State Analyzer (PSA) before being collected by the detector. The miniaturized PSG and PSA, inserted in the polarimetric box, are based on the use of bistable V-shaped ferroelectric liquid crystals (FLCs) in smectic C phase^49^. The FLCs operate as wave plates with fixed linear phase retardance and a variable azimuth of the fast (or slow) axis. In particular, the PSG is composed in order, taking into account the direction of light propagation, by a linear polarizer, a first FLC (FLC1), a waveplate (WP) and a second FLC (FLC2). The PSA is specular to the PSG. Indeed, it is composed of the same optical elements as the PSG but placed in the reverse order. A sketch of the polarimetric box, as well as a detailed description of the PSG and PSA are shown in Fig. 6b.

The PSG produces four polarization states described by four Stokes vectors that represent the columns of the real 16-component modulation matrix ${\mathbf{W}}$**.** After interaction with the sample, each polarization state produced by the PSG is analyzed by four polarization configurations of the PSA described by four Stokes vectors that represent the rows of the real 16-component analysis matrix ${\mathbf{A}}$**.** This procedure allows the acquisition of 16 intensity images which are grouped into the real 16-component intensity matrix ${\mathbf{B}}$ expressed as:

1 $${\mathbf{B}} = {\mathbf{AMW}}.$$

If ${\mathbf{A}}$ and ${\mathbf{W}}$ matrices have been previously determined by a calibration procedure, the Mueller matrix ${\mathbf{M}}$ of the cervical tissue can be obtained as:

2 $${\mathbf{M}} = {\mathbf{A}}^{ - 1} {\mathbf{BW}}^{ - 1} .$$

The relative orientations of the different components of the PSG and PSA are optimized so as to maximize the inverse of the condition number of the ${\mathbf{W}}$ and ${\mathbf{A}}$ matrices, in order to minimize the numerical error produced by their inversion, necessary to extract ${\mathbf{M}}$ using Eq. (2)^50^. The Eigenvalue Calibration Method is used to determine the ${\mathbf{W}}$ and ${\mathbf{A}}$ matrices^51^.

The MPC was equipped with a detection system to obtain the simultaneous acquisition of images at 550 and 650 nm, as shown in Fig. 6c. It consists of a first long-pass dichroic beam splitter (Thorlabs DMLP605) that spatially separates the upper part of the light spectrum above 605 nm and its lower part into two orthogonal beams. In addition, dichroic filters were placed in front of each of these two cameras to measure Mueller polarimetric images at either 650 nm for the camera "1" (Thorlabs FB650-40, 40 nm FWHM) or 550 nm for the camera "2" (Thorlabs FB550-40, 40 nm FWHM), with a spectral bandwidth of 40 nm for both wavelengths. The model of camera used for each wavelength is an Allied Prosilica GT1920 which provides 800 × 600-pixel images (in binning mode) with a frame rate of 25 images per second. Thus, the multispectral MPC allows the acquisition of reliable Mueller polarimetric images simultaneously at 550 nm and 650 nm in 1.6 s with a field of view of 4 × 3 cm^2^ and a real measured spatial resolution of about 100 µm/pixel. Furthermore, the images acquired for the two wavelengths are superimposable pixelwise.

### Clinical and measurement protocol

The results described in this work were obtained in a monocentric prospective observational pilot study conducted in a type 4 maternity unit of the Brugmann University Hospital in Brussels (Belgium). This study was approved by the Hospital Ethics Committee OM 026 of the Brugmann University Hospital. All research was conducted in accordance with the Declaration of Helsinki. Informed consent was obtained from all participants and/or their legal guardians.

The MPC was used to examine the cervix of 28 pregnant women of different gestational ages. Each patient was recruited and examined only once. Patients analyzed ranged in age from 16 to 48 years. Their gestational age ranged from 17 weeks of amenorrhea to 39 weeks of amenorrhea and 2 days. The patients included in this prospective study agreed to sign an informed consent. Mueller polarimetric images were acquired after installing a black speculum to minimize stray reflections. In addition, the date of childbirth was recorded for each patient.

This study focuses on examining pregnant women's cervix with full-term gestation. For this reason, only women who delivered at term were considered for the final analysis. In particular, four patients were excluded: three because they delivered prematurely (two of them had preventive cervical cerclage at the time of image acquisition) and the fourth because she had syphilis, which significantly alters the microstructure of the cervix, thus modifying its polarimetric response^52,53^. For the remaining 24 patients included in the statistical analysis, the age group did not change, while the gestational age ranged from 20 weeks of amenorrhea and 1 day to 39 weeks of amenorrhea and 2 days.

For each patient, cervical length was measured by transvaginal ultrasound after polarimetric imaging. Cervical length measurement by transvaginal ultrasound is performed according to a standardized procedure. A pregnant woman is asked to empty her bladder. The examination takes place in the gynecological position. The high-frequency vaginal probe, protected by a sterile sheath, covered with a gel, is inserted into the anterior vaginal fornix. A sagittal view of the cervix, showing the internal os, the cervical canal and the external os, is obtained on a screen. The practitioner measures the distance between the internal os and the external os which represents the cervical length. The pressure of the probe on the cervix should be as low as possible to obtain an accurate measurement. The duration of the procedure varies between 3 and 5 min.

### Data analysis

Mueller matrices of cervical tissue were first processed using symmetric decomposition to separate the main polarimetric properties, i.e., depolarization, retardance, and diattenuation^54^. Then, other depolarization metrics were explored in a second step, such as the depolarization index ${\text{P}}_{\Delta }$ ^55^**,** the polarimetric entropy ${\text{S}}$ ^56^, the purity indices ${\text{IPP}}_{1}$, ${\text{IPP}}_{2}$, and ${\text{IPP}}_{3}$ ^57^**,** the overall purity ${\text{PI}}$ ^57^**,** and the eigenvalues of the coherence matrix ${\uplambda }_{1}$, ${\uplambda }_{2}$, ${\uplambda }_{3}$, and ${\uplambda }_{4}$ ^58^**.**

#### Symmetric decomposition

This method allows the measured Mueller matrix ${\mathbf{M}}$ to be described as:

3 $${\mathbf{M}} = {\mathbf{M}}_{{{\text{D}}2}} {\mathbf{M}}_{{{\text{R}}2}} {\mathbf{M}}_{\Delta } {\mathbf{M}}_{{{\text{R}}1}}^{t} {\mathbf{M}}_{{{\text{D}}1}}$$

where ${\mathbf{M}}_{{{\text{D}}i}}$ $\left( {i = 1,2} \right)$ represent two diattenuators, ${\mathbf{M}}_{{{\text{R}}i}}$ $\left( {i = 1,2} \right)$ two retarders and ${\mathbf{M}}_{\Delta }$ a diagonal depolarizer. Symmetric decomposition is a priori particularly relevant for the study of biological tissues where the most superficial layers produce diattenuation and retardance effects when light enters and leaves the sample. In contrast, volume scattering produces diagonal depolarization.

The most relevant polarimetric properties observed on cervical tissue are: (i) the total depolarization $\Delta$ and (ii) the linear birefringence characterized by the phase retardance ${\text{R}}$ and the azimuth ${\upalpha }$.

The parameter $\Delta$ can be obtained using the following expression:

4 $$\Delta { } = 1 - \frac{{\left| {{\text{M}}_{\Delta 22} } \right| + \left| {{\text{M}}_{\Delta 33} } \right| + \left| {{\text{M}}_{\Delta 44} } \right|}}{3}$$

where ${\text{M}}_{\Delta 22}$, ${\text{M}}_{\Delta 33}$, and ${\text{M}}_{\Delta 44}$ are the diagonal components of ${\text{M}}_{\Delta }$. This parameter ranges from 0 to 1 for a non-depolarizing or a pure depolarizing sample, respectively.

The parameter ${\text{R}}$ is obtained using the following expression:

5 $${\text{R}} = {\text{arccos}}\left[ {\frac{{{\text{tr}}\left( {{\text{M}}_{{\text{R}}} } \right)}}{2} - 1} \right]$$

where ${\text{tr}}\left( {{\mathbf{M}}_{{\text{R}}} } \right)$ is the trace of ${\mathbf{M}}_{{\text{R}}} = {\mathbf{M}}_{{{\text{R}}2}} {\mathbf{M}}_{{{\text{R}}1}}^{t}$ (${\mathbf{M}}_{{{\text{R}}1}}^{t}$ is the transposed matrix of ${\mathbf{M}}_{{{\text{R}}1}}$). It is measured in degrees and generally varies between 0° and 180° for biological tissues.

The parameter ${\upalpha }$ is obtained using the following expression:

6 $${\upalpha } = {\text{arctan}}\left[ {\frac{{{\text{M}}_{{{\text{R}}24}} }}{{{\text{M}}_{{{\text{R}}43}} }}} \right]$$

which varies between − 45° and + 45°. This range is extended between 0° and 180° following the procedure described in^42,43^ in order to reduce the recurring jumps in the colormap of the images corresponding to ${\upalpha }$, thus simplifying their visual interpretation.

#### Depolarization index

It is a single-number metric that provides an average measure of the depolarizing power of a sample. This parameter is defined as:

7 $${\text{P}}_{\Delta } = \sqrt {\frac{{{\text{tr}}\left( {{\mathbf{M}}^{{\text{t}}} {\mathbf{M}}} \right) - {\text{M}}_{11}^{2} }}{{3{\text{M}}_{11}^{2} }}}$$

where the ${\text{M}}_{11}$ component of ${\mathbf{M}}$ is the unpolarized intensity image. ${\text{P}}_{\Delta }$ is a non-dimensional parameter invariant to the change of reference frame for the Mueller matrix measurement. Its value ranges from 0 for a fully depolarizing sample to 1 for a non-depolarizing sample.

#### Eigenvalues of coherence matrix

The eigenvalues of the covariance matrix ${\uplambda }_{1} \ge {\uplambda }_{2} \ge {\uplambda }_{3} \ge {\uplambda }_{4}$ are themselves suitable depolarization metrics. The covariance matrix ${\text{H}}$ is given by:

8 $${\text{H}} = \mathop \sum \limits_{{{\text{i}},{\text{j}} = 1}}^{4} {\text{M}}_{{{\text{ij}}}} \left( {{\upsigma }_{{\text{i}}} \otimes {\upsigma }_{{\text{j}}}^{*} } \right)$$

where ${\upsigma }_{{\text{i}}} { }\left( {i = 1,2,3} \right)$ are the Pauli matrices, ${\text{M}}_{{{\text{ij}}}}$ (${\text{i}},{\text{j}} = 1,2,3,4$) are the coefficients of ${\mathbf{M}}$ and $\otimes$ is the Kronecker product.

#### Polarimetric entropy

It is a scalar parameter that quantifies the degree of disorder in a sample**.** This parameter is defined as:

9 $${\text{S}} = - \mathop \sum \limits_{{{\text{i}} = 1}}^{4} {\uplambda }_{{\text{i}}} {\text{log}}_{4} {\uplambda }_{{\text{i}}}$$

where ${\uplambda }_{{\text{i}}} { }\left( {{\text{i}} = 1,2,3,4} \right)$ are the eigenvalues of the covariance matrix ${\text{H}}$ (Eq. 8) of the measured Mueller matrix ${\mathbf{M}}$**.**

#### Indices of polarimetric purity

The analysis of the different possible depolarization sources has led to the introduction of polarimetric purity indices that are three different coefficients indicated as ${\text{IPP}}_{1}$, ${\text{IPP}}_{2}$, and ${\text{IPP}}_{3}$ respectively. These coefficients are defined as:

10 $${\text{IPP}}_{1} = \frac{{{\uplambda }_{1} - {\uplambda }_{2} }}{{{\text{tr}}\left( {\text{C}} \right)}}$$

11 $${\text{IPP}}_{2} = \frac{{{\uplambda }_{1} + {\uplambda }_{2} - 2{\uplambda }_{3} }}{{{\text{tr}}\left( {\text{C}} \right)}}$$

12 $${\text{IPP}}_{3} = \frac{{{\uplambda }_{1} + {\uplambda }_{2} + {\uplambda }_{3} - 3{\uplambda }_{4} }}{{{\text{tr}}\left( {\text{C}} \right)}}$$

where ${\uplambda }_{1} \ge {\uplambda }_{2} \ge {\uplambda }_{3} \ge {\uplambda }_{4}$ are the eigenvalues of the covariance matrix ${\text{H}}$ (Eq. 8) of ${\mathbf{M}}$. The purity indices can be combined to obtain the overall purity index PI, given by:

13 $${\text{PI}} = \sqrt {\frac{{{\text{IPP}}_{1}^{2} + {\text{IPP}}_{2}^{2} + {\text{IPP}}_{3}^{2} }}{3}}$$

## Supplementary Information

Supplementary Information.

## Data Availability

All data generated or analyzed during this study are included in this published article [and its supplementary information files].
