# Supplementary material for: Depolarization imaging for fast and non-invasive monitoring of cervical microstructure remodeling in vivo during pregnancy
Source: Sci Rep. 2022 Jul 19;12:12321. doi: 10.1038/s41598-022-15852-w (PMC9296502; doi:10.1038/s41598-022-15852-w)
Supplement: Supplementary file 1 — Supplementary Information. [file 41598_2022_15852_MOESM1_ESM.docx]

Depolarization imaging for fast and non-invasive monitoring of cervical microstructure remodeling *in vivo* during pregnancy

Jean Rehbinder^2¶^, Jérémy Vizet^1¶^, Junha Park^1^, Razvigor Ossikovski^1^, Jean-Charles Vanel^1^, André Nazac^3^, Angelo Pierangelo^1^*

^1^LPICM, CNRS, Ecole polytechnique, IP Paris, Palaiseau, 91128, France

^2^ICube, CNRS, Université de Strasbourg, 67412 Illkirch cedex, France

^3^Department of Gynaecology, Iris Sud Ixelles Hospital, 1050 Ixelles, Belgium

*, corresponding author, Angelo Pierangelo

LPICM, CNRS, Ecole polytechnique, IP Paris, Palaiseau, 91128, France

+33169334369

*angelo.pierangelo@polytechnique.edu*

^¶^, equal contributions, J.R. and J.V. contributed equally to this work

Appendix


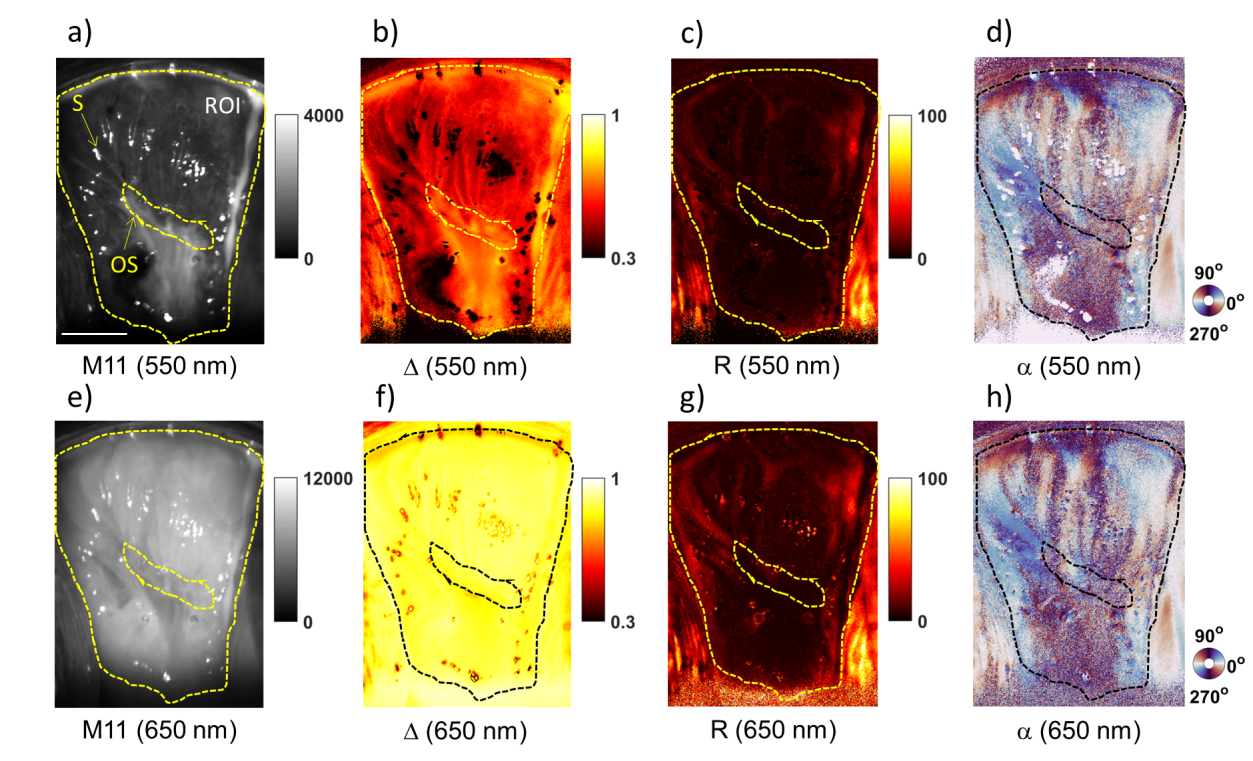


**Figure S1.**a), e), Monochromatic unpolarized intensity image of P2's ectocervix corresponding to the unnormalized coefficient $M_{11}$ of the measured Mueller matrix at 550 and 650 nm, respectively. b), f) Total depolarization image $\Delta$ at 550 and 650 nm, respectively. c), g) Linear phase retardance image $R$ at 550 and 650 nm, respectively. d), h) Azimuth image $\alpha$ at 550 and 650 nm, respectively. In a), the white bar corresponds to 1 cm, OS indicates the pixels in the region of the external os, and S indicates the pixels saturated by specular reflections.


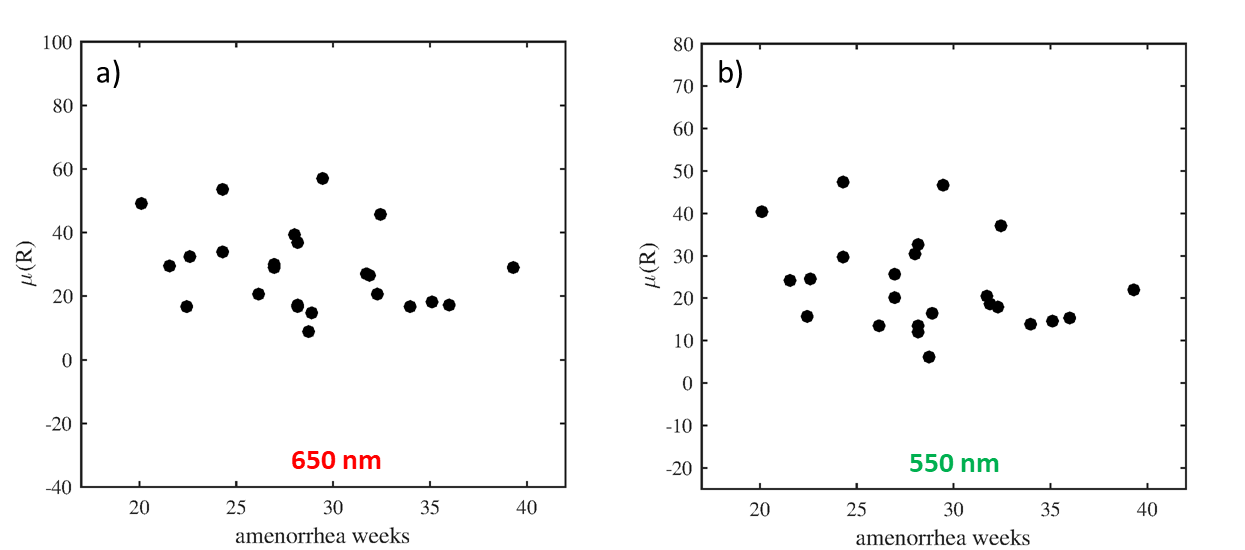


**Figure S2.**a), b) Evolution curves describing the modification of $\mu(R)$ according to the gestational age at 650 nm and 550 nm, respectively. This parameter is not correlated with gestational age for both wavelengths.

**
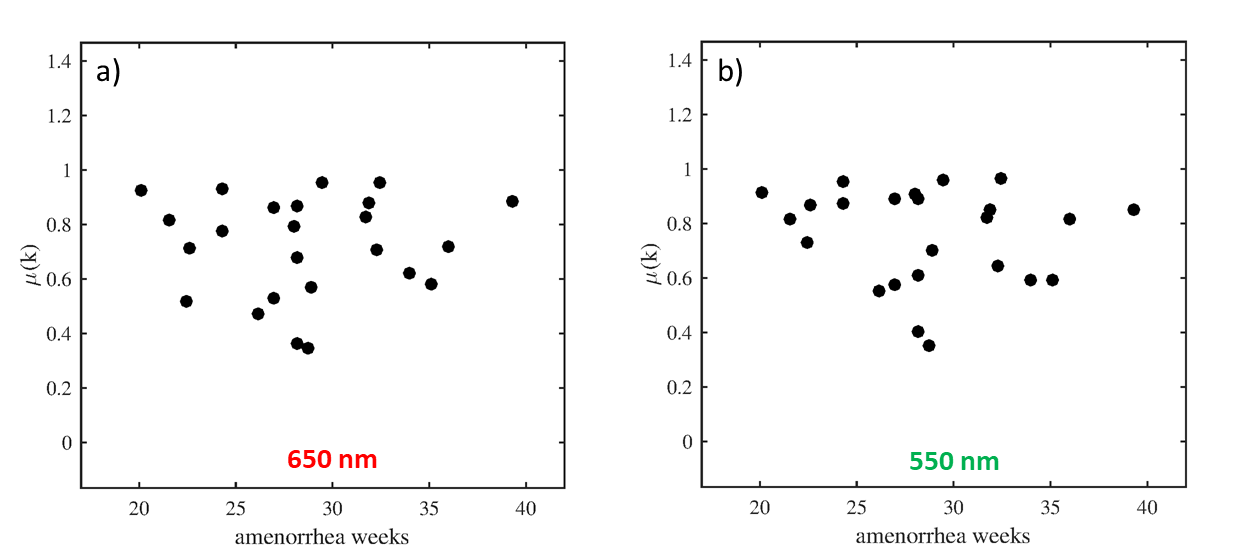
**

**Figure S3.**a), b) Evolution curves describing the modification of $\mu(k)$ according to the gestational age at 650 nm and 550 nm, respectively. This parameter is not correlated with gestational age for both wavelengths.

**
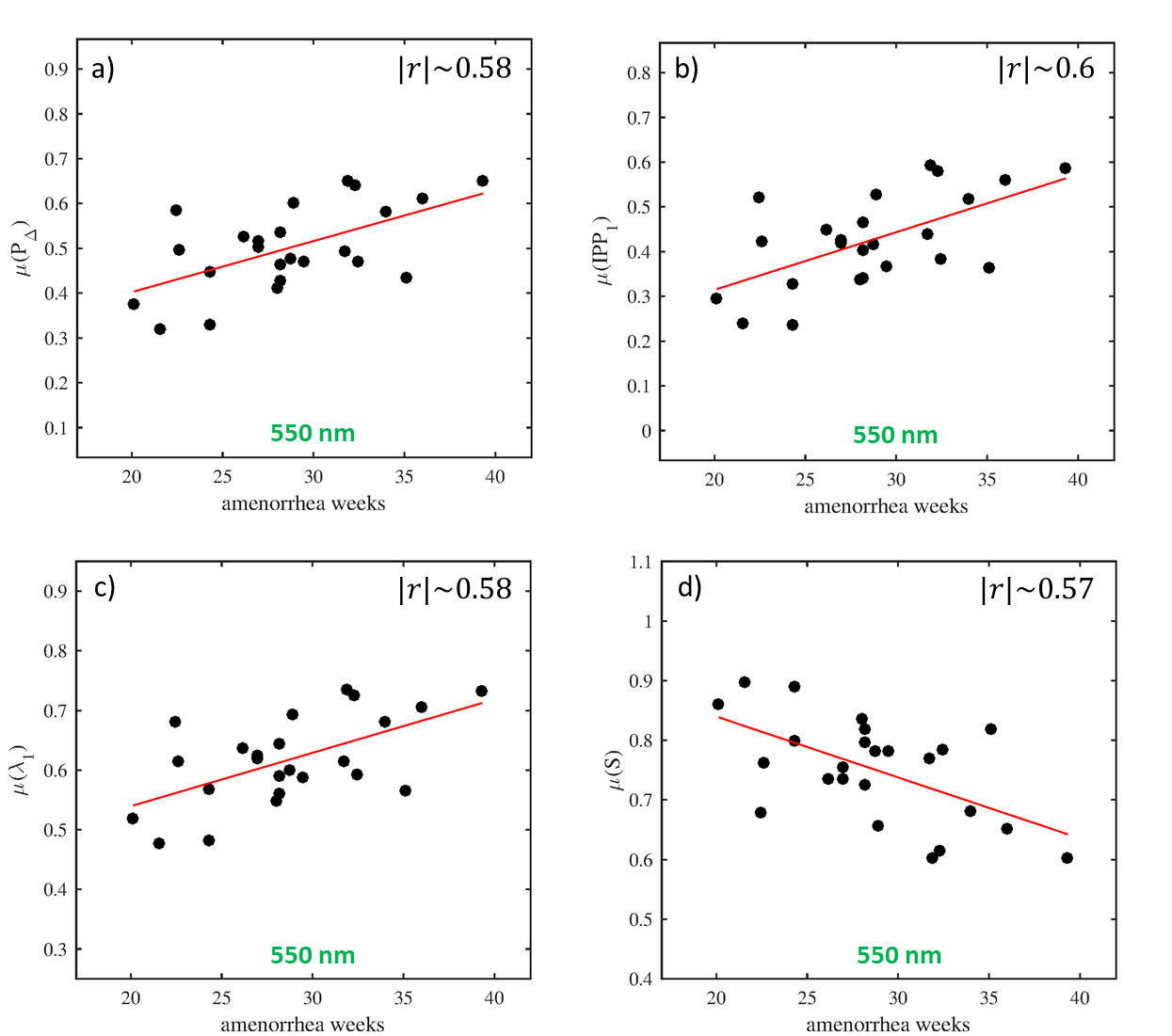
**

**Figure S4.** a), b), c), d) Evolution curves describing the modification of $\mu(P_{\Delta})$, $\mu(\mathrm{IPP}_{1})$, $\mu(\lambda_{1})$, and $\mu(S)$ at 550 nm according to the gestational age, respectively. These parameters have a moderate correlation with the gestational age, $\left| r \right|$ varying between $0.57$ and $0.6$

**
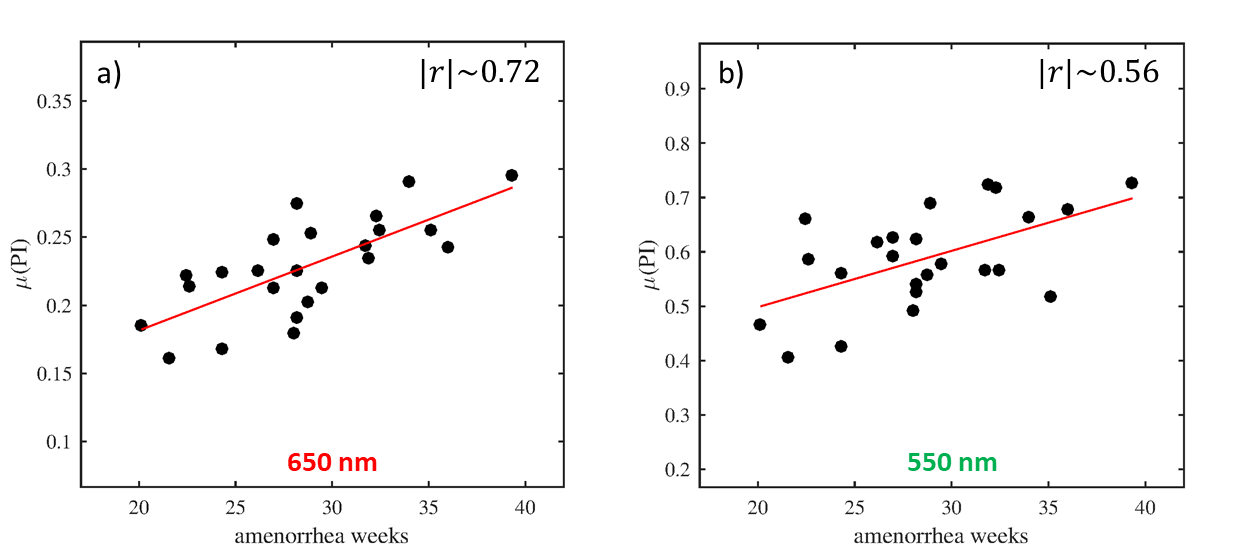
**

**Figure S5.** a), b) Evolution curves describing the modification of $\mu(PI)$ according to the gestational age at 650 nm and 550 nm, respectively. The linear correlation of this parameter with the gestational age is high at 650 nm ($\left| r \right|\sim0.72$) and moderate at 550 nm ($\left| r \right|\sim0.56$).


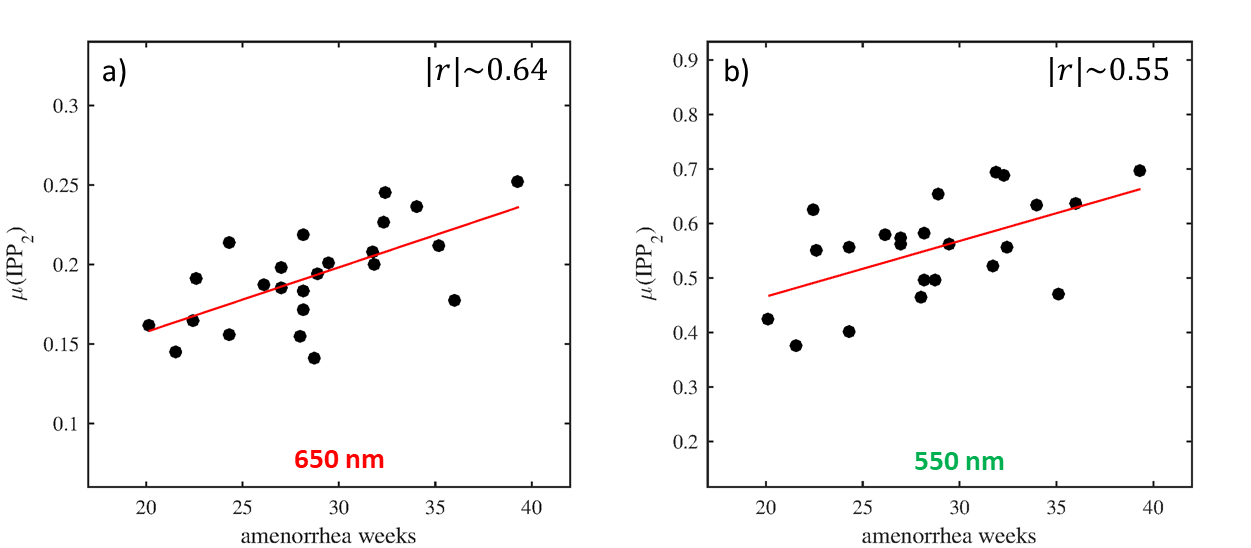


**Figure S6.** a), b) Evolution curves describing the modification of $\mu(\mathrm{IPP}_{2})$ according to the gestational age at 650 nm and 550 nm, respectively. The linear correlation of this parameter with the gestational age is moderate at 650 nm ($\left| r \right|\sim0.64$) and 550 nm ($\left| r \right|\sim0.55$).


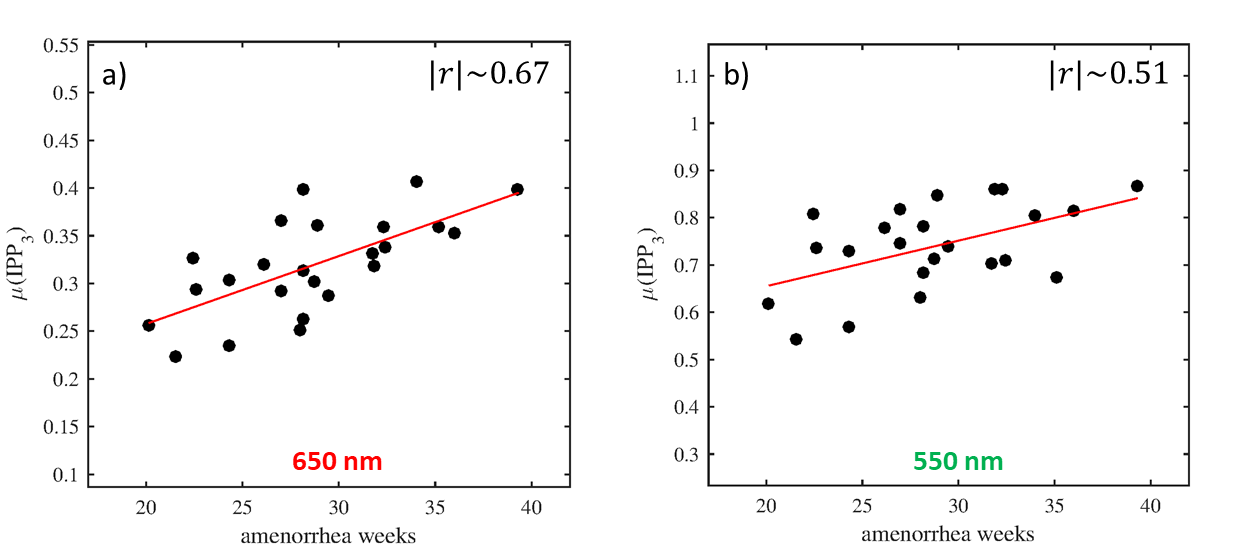


**Figure S7.** a), b) Evolution curves describing the modification of $\mu(\mathrm{IPP}_{3})$ according to the gestational age at 650 nm and 550 nm, respectively. The linear correlation of this parameter with the gestational age is moderate at 650 nm ($\left| r \right|\sim0.67$) and 550 nm ($\left| r \right|\sim0.51$).


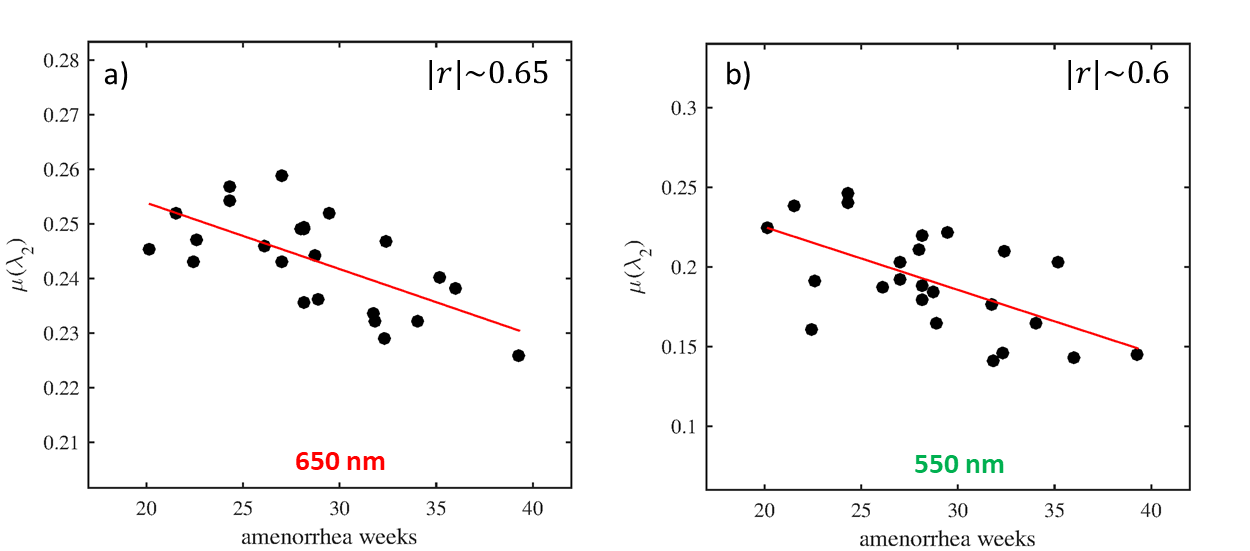


**Figure S8.** a), b) Evolution curves describing the modification of $\mu(\lambda_{2})$ according to the gestational age at 650 nm and 550 nm, respectively. The linear correlation of this parameter with the gestational age is moderate at 650 nm ($\left| r \right|\sim0.65$) and 550 nm ($\left| r \right|\sim0.6$).


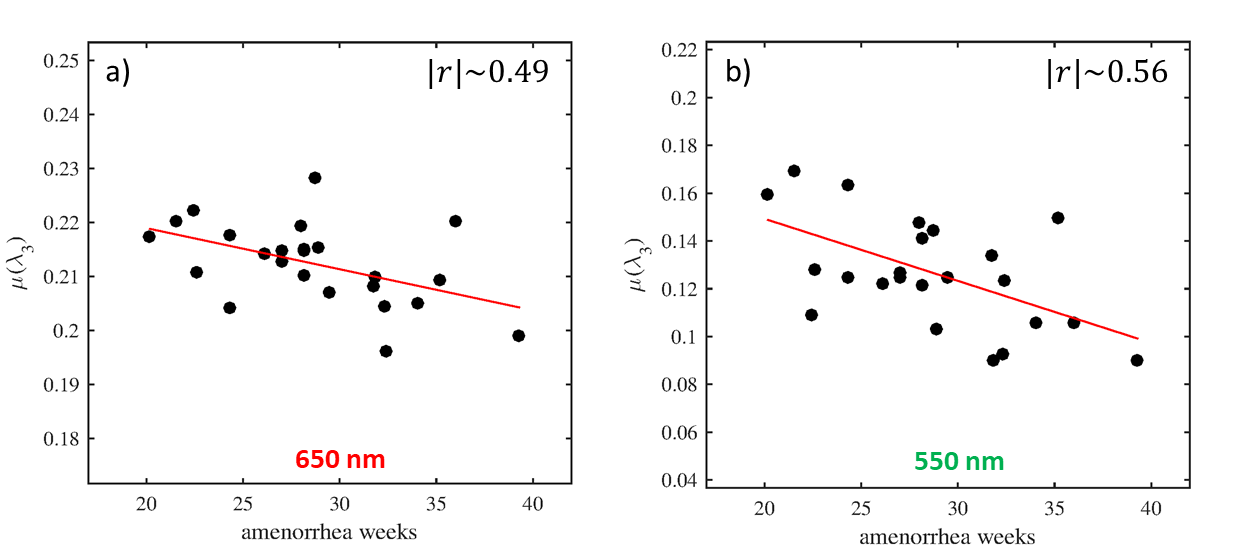


**Figure S9.** a), b) Evolution curves describing the modification of $\mu(\lambda_{3})$ according to the gestational age at 650 nm and 550 nm, respectively. The linear correlation of this parameter with the gestational age is moderate at 650 nm ($\left| r \right|\sim0.49$) and 550 nm ($\left| r \right|\sim0.56$).


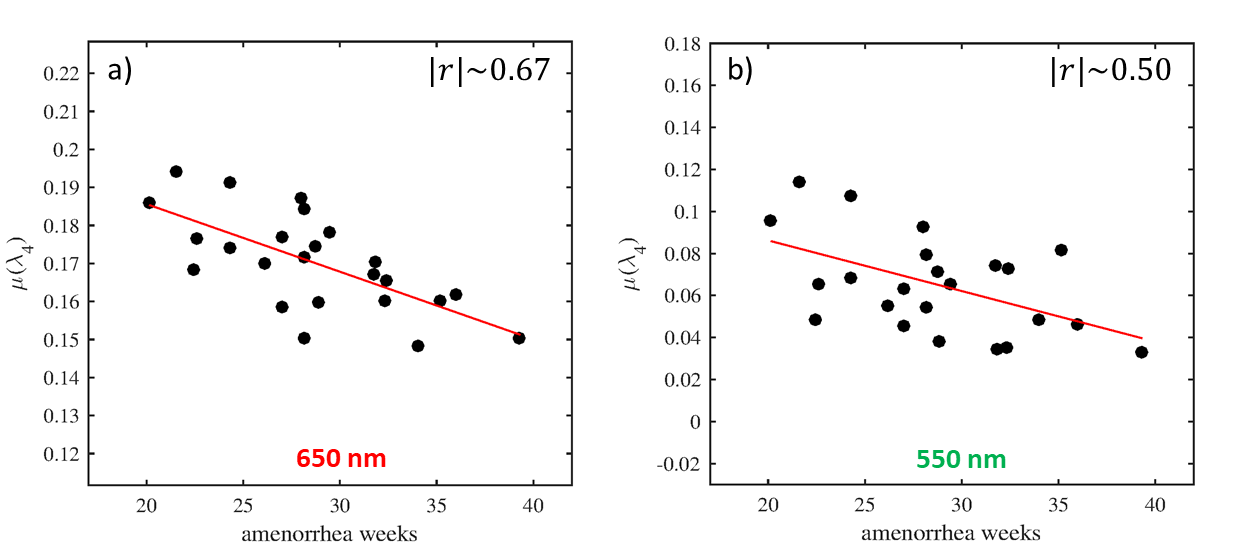


**Figure S10.** a), b) Evolution curves describing the modification of $\mu(\lambda_{4})$ according to the gestational age at 650 nm and 550 nm, respectively. The linear correlation of this parameter with the gestational age is moderate at 650 ($\left| r \right|\sim0.67$) and 550 nm ($\left| r \right|\sim0.50$).
